# Editorial: Hunger, Food Sovereignty and Public Health

**DOI:** 10.3389/ijph.2025.1608367

**Published:** 2025-02-21

**Authors:** Janeth Mosquera, Lyda Osorio

**Affiliations:** Epidemiology and Population Health Research Group (GESP), Faculty of Health, School of Public Health, Universidad del Valle, Cali, Colombia

**Keywords:** hunger, food security, public health, climate change, vulnerable populations

The escalating environmental and social crises have long warned of worsening challenges related to food security and sovereignty [1]. However, the COVID-19 pandemic revealed the fragility of food systems across geographic scales. In Latin America, 247.8 million people in 2022 were forced to reduce the quality or quantity of their food, experienced hunger, or went days without eating [2]. The current climate crisis has emerged as a significant public health challenge due to its direct and indirect impacts on various dimensions of wellbeing. Notably, the second goal of the 2030 Agenda for Sustainable Development Goals— “Zero Hunger”— increasingly appears unattainable, partly because of climate change [3]. Unsurprisingly, the impacts of climate change are unevenly distributed across regions and social groups. For example, 95% of crops in Africa rely on rainfall for irrigation, making the continent particularly vulnerable to climate variability, such as unpredictable rainfall patterns. Conversely, California irrigates 90% of its crops with advanced technology, yet much of this agricultural production is industrial [4].

Hunger is not merely an environmental or technical issue—it is a political one. Decisions made by those in power directly affect what families can place on their tables daily. While technological innovations play a role in increasing food production, addressing hunger requires broader measures. These include ensuring adequate and sufficient land for food cultivation (e.g., agrarian reform), promoting agroecological models that counter industrial monocultures, protecting marine ecosystems, and advancing fair trade in food markets. States must also confront violence against environmental leaders and improve public awareness, as misinformation about the climate crisis, its drivers, and the role of the agribusiness industry continues to spread through mass media and social networks [5].

In response to these challenges, international technical cooperation agencies have proposed various agendas to mitigate global hunger. Meanwhile, communities persist in their resistance and develop collaborative initiatives to ensure food security in rural and urban areas. For example, La Vía Campesina continues to advocate for food sovereignty, while agroecological family farming initiatives are gaining momentum in rural Colombia [6].

This Special Issue features seven papers that explore diverse strategies and innovations aimed at addressing the multifaceted challenges of hunger and public health. They highlight how to integrate food insecurity into routine clinical care to improve risk assessment and management in patients Choe and Pak; examine the unique challenges of food insecurity faced by older women, less educated individuals and those in impoverished geographic areas in Thailand Phulkerd et al.; demonstrate that food bank users in Canada exhibit diverse strategies to cope with food insecurity Pérez et al., show how community initiatives (e.g., communal kitchens) proved more effective than food distribution platforms in addressing hunger and fostering social solidarity during the COVID-19 lockdown in Colombia Chavarro and Mosquera-Becerra; call for a paradigm shift from viewing recipients of nutrition programs as beneficiaries to acknowledging them as human rights holders and policy allies Mejía Toro et al.; and propose evidence-based strategies to eradicate hunger Arigbede et al. Additionally, these papers serve as examples of measuring food insecurity and other relevant methods.

The current food landscape is undeniably complex and demanding. Despite significant progress over recent decades, hunger remains a grim reality for millions. The guiding principle for addressing this challenge must be the universal right to food. Achieving this requires medium-term investments in early warning and rapid response systems to prevent and mitigate food crises, alongside long-term local and international actions grounded in environmentally respectful agroecological agriculture and the production of nutrient-rich foods. Let us strive for a world where food security is not a privilege but a fundamental right, achieved through collective commitment, equity, and sustainable practices.
